# Availability and Accessibility of Primary Care for the Remote, Rural, and Poor Population of Indonesia

**DOI:** 10.3389/fpubh.2021.721886

**Published:** 2021-09-21

**Authors:** Supriyatiningsih Wenang, Juergen Schaefers, Andi Afdal, Ali Gufron, Siegfried Geyer, Iwan Dewanto, Joerg Haier

**Affiliations:** ^1^Department of Pediatric Oncology, University Hospital Muenster, Muenster, Germany; ^2^Department of Obstetrics and Gynecology, Faculty of Medicine and Health Sciences, University of Muhammadiyah Yogyakarta, Bantul, Indonesia; ^3^IGP Institute for Health Sciences and Public Health, Muenster, Germany; ^4^Comprehensive Cancer Center Hannover, Hannover Medical School, Hannover, Germany; ^5^Badan Penyelenggara Jaminan Sosial (Social Insurance Administration Organization), Jakarta, Indonesia; ^6^Institute for Sociology, Hannover Medical School, Hannover, Germany; ^7^Faculty of Medicine and Health Sciences, School of Dentistry, University of Muhammadiyah Yogyakarta, Bantul, Indonesia

**Keywords:** healthcare delivery, poor population, primary care, remote population, availability, accessibility, acceptability, universal health coverage

## Abstract

**Background:** Adopting Universal Health Coverage for implementation of a national health insurance system [Jaminan Kesehatan Nasional (JKN)/Badan Penyelenggara Jaminan Sosial or the Indonesian National Social Health Insurance Scheme (BPJS)] targets the 255 million population of Indonesia. The availability, accessibility, and acceptance of healthcare services are the most important challenges during implementation. Referral behavior and the utilization of primary care structures for underserved (rural/remote regions) populations are key guiding elements. In this study, we provided the first assessment of BPJS implementation and its resulting implications for healthcare delivery based on the entire insurance dataset for the initial period of implementation, specifically focusing on poor and remote populations.

**Methods:** Demographic, economic, and healthcare infrastructure information was obtained from public resources. Data about the JKN membership structure, performance information, and reimbursement were provided by the BPJS national head office. For analysis, an ANOVA was used to compare reimbursement indexes for primary healthcare (PHC) and advanced healthcare (AHC). The usage of primary care resources was analyzed by comparing clustered provinces and utilization indices differentiating poor [Penerima Bantuan Iur (PBI) membership] and non-poor populations (non-PBI). Factorial and canonical discrimination analyses were applied to identify the determinants of PHC structures.

**Results:** Remote regions cover 27.8% of districts/municipalities. The distribution of the poor population and PBI members were highly correlated (*r*^2^ > 0.8; *p* < 0.001). Three clusters of provinces [remote high-poor (*N* = 13), remote low-poor (*N* = 15), non-remote (*N* = 5)] were identified. A discrimination analysis enabled the >82% correct cluster classification of infrastructure and human resources of health (HRH)-related factors. Standardized HRH (nurses and general practitioners [GP]) availability showed significant differences between clusters (*p* < 0.01), whereas the availability of hospital beds was weakly correlated. The usage of PHC was ~2-fold of AHC, while non-PBI members utilized AHC 4- to 5-fold more frequently than PBI members. Referral indices (*r*^2^ = 0.94; *p* < 0.001) for PBI, non-PBI, and AHC utilization rates (*r*^2^ = 0.53; *p* < 0.001) were highly correlated.

**Conclusion:** Human resources of health availability were intensively related to the extent of the remote population but not the numbers of the poor population. The access points of PHC were mainly used by the poor population and in remote regions, whereas other population groups (non-PBI and non-Remote) preferred direct access to AHC. Guiding referral and the utilization of primary care will be key success factors for the effective and efficient usage of available healthcare infrastructures and the achievement of universal health coverage in Indonesia. The short-term development of JKN was recommended, with a focus on guiding referral behavior, especially in remote regions and for non-PBI members.

## Introduction

Since the year 2000, many countries have adopted the concept of universal health coverage as a strategic background for their national policies, thus inducing the implementation of reforms, particularly in low- and middle-income countries. The evaluation and quality assurance of healthcare delivery goals (availability, accessibility, affordability, acceptability, and quality of care) are typically defined by three dimensions: covered population (pooled funds–solidarity principle), healthcare infrastructure, and healthcare services covered by healthcare funds (1).

Perhaps one of the most ambitious examples for this is the national health scheme being implemented in Indonesia [*Jaminan Kesehatan Nasional* (JKN)], which made healthcare available to its 255 million population, subsequently becoming the largest single healthcare system worldwide (2). This governmental approach has been intensively changing the expectations of the enrolled population, the required healthcare processes such as referral systems, and treated morbidity patterns. However, very specific challenges have originated from the size, diversity of urban, rural, and remote environments, variable levels of socioeconomic developments within the country, and the growing number of metropolitan areas of Indonesia. Furthermore, an epidemiological transition has produced a double burden of diseases for Indonesia, with the simultaneous increase of non-communicable diseases, such as diabetes, cerebrovascular disease, and ischemic heart disease, while infectious causes, such as tuberculosis, diarrhea, and HIV/AIDS, persist as substantial problems (3). Thus, starting in 2014, the Indonesian National Social Health Insurance Scheme [*Badan Penyelenggara Jaminan Sosial* (BPJS)] (4) aimed to provide healthcare coverage for all Indonesians. For the poor and near-poor population, insurance fees were waved totally or in part (PBI membership). As a result, enrollment in the program increased from 86.4 million (2014) to 111.6 million (2017) members, with the program securing additional funds at the national (92.2 million) or local levels (19.4 million) (5). Thus, BPJS healthcare insurance became mandatory for all Indonesian people as of 2019 (6). Since then, patients have no longer been requested to handle reimbursement issues, as the financial flow has been directly preceded between healthcare providers and BPJS/the government. Furthermore, healthcare infrastructure in Indonesia for primary care is mainly based on puskesmas (outpatients) and primary care hospitals (class A), whereas advanced care is mostly provided in level B/C (secondary/tertiary) hospitals and at the national level (class D hospitals). The reimbursement system differentiated between the Fasilitas Kesehatan Tingkat Pertama (FKTP) for primary healthcare (PHC) and the Fasilitas Kesehatan Rujukan Tingkat Lanjut (FKRTL) for advanced healthcare (AHC) and referral services.

During the BPJS roll-out, different participant groups did not enroll simultaneously. Furthermore, the initial proportion of the PBI members that did not receive subsidiaries was higher than their respective proportion within the population of the country (7, 8). This suggested the potential consequences for healthcare process development and referral strategies similar to other countries (9, 10). Ford et al. (11) emphasized that socioeconomically disadvantaged people living in rural areas face various barriers that limit their access to primary care. However, experiences and solutions of healthcare insurance implementation cannot be easily transferred between various countries (12).

Primary care is recognized as the most important form of healthcare for maintaining population health because it is relatively inexpensive, can be more easily delivered than specialty and inpatient care, and, if properly distributed, it is most effective in preventing disease progression on a wide scale. Recent advances in the field of health geography have greatly improved the understanding of the role played by the geographic distribution of health services in population health maintenance. However, most of this knowledge has been accrued for hospital and specialty services in rural areas (13). Previous evaluations for Indonesia suggested that the inequity of healthcare usage currently occurs, where poor populations and those living in remote areas would benefit less due to their limited geographical access to primary health care in particular. For instance, allocating physicians to remote islands or in mountainous or forest locations subsequently resulting in a shortage of essential health workers have been recognized as a major challenge (14, 15). Financial protection through social health insurance can be provided, but access among poor and rural/remote populations has remained an issue. Therefore, in Indonesia, PHC provision is important for the assessment of the establishment of a structured improvement process as part of the healthcare insurance roll-out.

This study was the first assessment of BPJS implementation and its resulting implications for healthcare delivery based on the entire insurance dataset for the initial period of implementation. The analysis included all 34 provinces and focused on the differences between rural/remote and urban and between poor and non-poor populations (16, 17). Based on the demographic, reimbursement, and membership data, the usage of healthcare resources with a specific focus on underserved populations (remote regions and poor populations, according to governmental definition) was analyzed. Since preliminary observations suggested inefficient referral structures the utilization of primary care structures compared to advanced care in comparison between various insurance groups was an additional focus. Furthermore, the relationships between the current availability of healthcare resources and their usage were evaluated. The bridging aim was to support strategic decisions for the development of healthcare resources and delivery processes as a differentiated approach for the provinces of Indonesia.

## Methods

### Study Design

#### Data Acquisition

Demographic data, economic parameters, and healthcare infrastructure information were obtained from the public resources of the national statistical agency (18). Data about the JKN membership structure, performance information, and reimbursement (differentiated for PHC and AHC) were provided by the BPJS national head office. Data for the entire year of 2018 were used for the analysis. These data were provided as a random selection from a family membership-based JKN registration (~73.4 member families). For each sample data, an individual sampling weight was calculated and used in SPSS calculations (a detailed description of the data extraction is provided as Supplementary File 1). If applicable, data were normalized for population density (per 1,000) and human resource distribution (per unit). A list and the corresponding descriptions of all the used parameters are available as Supplementary Table 1. Ethical approval was obtained from the Muhammaniyah University (No. 202/EC-KEPK FKIK UMY/Vlll/2020) and the Indonesian National Healthcare Insurance BPJS (No. 5060/I.2/0419) for the entire project. Informed consent was not applicable in this type of investigation.

#### Study Setting

Members of the JKN national health insurance were divided into two major groups:

- poor people supported due to low income (PBI memberships);
- members not supported regarding insurance fees (non-PBI memberships).

Healthcare provision was distinguished into two different groups: PHC for primary healthcare (*puskemas*, independent general practitioners [GP], and class A hospitals) and AHC for advanced care in class B/C/D hospitals. Furthermore, reimbursement by BPJS consists of primary care capitation fees per insured member assigned to the healthcare provider (PHC capitation), procedure/diagnosis-related fees for primary care (PHC non-capitation), and reimbursements for advanced care according to respective efforts (AHC). Due to the lack of detailed data for healthcare procedures in a capitation-based system, the reimbursement parameters were used as indices for healthcare usage in different provider environments.

#### Definition of Underserved Regions

Indonesia has remote regions (*tertinggal* = underdeveloped, *terdepan*/*terjauh* = remote, and *terluar* = frontier/outer) consisting of 143 districts/municipalities at 27 provinces (from 34 provinces) defined based on governmental rules (19). This annotation was used for the analysis of the rural and remote populations (further referred to as Remote regions). According to this governmental nomenclature, information regarding the demographic characteristics of each province were available, such as the number of people in the (poor) population in these regions.

All statistical analyses were performed using IBM SPSS Statistics Version 26.

### Multivariate Analysis

#### Analytical Strategy

First, the reduction of high numbers of variables describing available healthcare resources was required. For the analysis of underserved populations and primary care-related healthcare delivery variables were combined using factorial analysis. Subsequently, for the differentiation of provinces with similar structures regarding poor and remote populations, cluster analysis was performed. Combining these descriptive approaches, the relationships between healthcare resources, delivery processes, and clustered provinces were evaluated. Finally, the usage of healthcare resources was analyzed by comparing clustered provinces, JKN membership groups, and primary vs. advanced care utilization indices.

Gross domestic product and parameters describing infrastructure and human resources in the provinces were considered as independent variables. In contrast, variables related to healthcare services (reimbursement data) were treated as dependent during multivariate analyses. For comparability between provinces, variables were calculated based on population size and standardized whenever possible.

#### Cluster Analysis

A cluster analysis was performed to identify groups of provinces for which developmental strategies of healthcare resources and delivery processes can be formulated. Demographic and economic characteristics were postulated as determinants of healthcare usage, particularly for vulnerable groups. The vulnerability was assumed for the poor population and groups living in remote regions. To group the provinces according to the low and high prevalence of vulnerable groups, clustering based on various parameters describing the mentioned vulnerability was applied (parameter 5–20 in Supplementary Table 1). According to the targeted differentiation between these groups, clustering was performed using partitioning approaches. The selection of the clustering method was driven by the need to minimize heterogeneity within a reasonable number of groups for further analyses. The stability of cluster annotation was ensured using two different clustering approaches (k-means and ward-methods). Cluster quality was accepted if both methods provided >90% coherence. To further test the contribution of the included variables for cluster segmentation, a discriminant analysis was applied. A univariate ANOVA and Eigenwert provided information about the quality of the discrimination functions.

#### Multivariate Factorial Analysis

Due to a large number of potential variables (parameters 42–206 in Supplementary Table 1) influencing healthcare utilization, we were required to reduce the observed complexity and aggregate the mentioned variables, if possible, into a limited number of factors that can be used for further analysis. Therefore, a multivariate factorial analysis was performed as a principal component analysis (PCA). The parameters were combined in a stepwise approach to finding out if a sufficient set of variables could describe available healthcare resources. Since some of the independent variables were, in part, interlinked with potential collinearity, an initial Pearson correlation was performed. As a result, a sufficient number of significant correlations was identified, suggesting eligibility for factorial analysis that was also approved by the Kaiser-Meyer-Olkin criterion (KMO, accepted if >0.5). The significance was also approved by a Bartlett test for sphericity.

#### ANOVA

The univariate group comparison was performed by an ANOVA method. For the multivariate analysis, *post-hoc* tests were done using the Scheffé procedure and Bonferroni correction. This was used for the analysis of the healthcare delivery parameters (reimbursement data: parameters 207–282 in Supplementary Table 1).

#### Healthcare Usage Rates

Non-capitation-based JKN reimbursement data enabled the differentiation of these groups, subsequently providing a robust data structure for the analysis of the utilization behavior of different JKN groups. To ensure the comparability of these data between the provinces, various indices were defined as the PHC utilization rate (# of non-capitation/# of capitation), JKN service rates [all non-capitation (PHC + AHC) services/# of the insured population], AHC utilization rate (AHC/# of the insured population), and referral index of primary to advanced care (AHC/non-capitation PHC). These parameters did not completely have the same background within the BPJS dataset. However, they were fully comparable between the provinces and the population groups. Therefore, they were used as indicators for the proposed delivery processes.

For the indicator for actual primary care service intensity, a PHC utilization index (= PHC Non-Capitation Service/PHC Capitation Service) was provided. The mentioned referral index of primary to advanced care was expressed by non-capitation AHC/PHC services. If appropriate, Student's *t*-tests were used for the comparison of these two groups. A canonical correlation analysis was done to address the distribution of the variances of the target parameters.

## Results

### Membership Development and Remote Regions

Enrollment into BPJS membership differed between various population groups. The percentage of PBI members declined from 71.4% in 2014 to 61.2% in 2017. On the other hand, non-PBI participants rose from 25 to 36% (Supplementary Table 2).

Of all the districts/municipalities, 27.8% belonged to remote regions. The overall HRH availability in primary care in the remote regions was approximately 170,700 healthcare workers, representing 14.4% of the total national HRH (16.4% nurses/midwives; 10.2% GP; 8.8% all physicians). The distribution of the overall poor population and JKN participants co-financed by the government (PBI groups) were highly correlated (*r*^2^ > 0.8; *p* < 0.001). In differentiating the remote and non-remote population, the correlation was slightly less for each subgroup but still highly significant (*r*^2^ > 0.6; *p* < 0.001).

Demographic variables describing the poor and remote populations for each province showed high correlations (Supplementary Table 3A). Insufficient KMO/Bartlett tests also supported the application of the variables “percentage of remote population” and “percentage of total poor in total remote population” as appropriate population descriptors for these two aspects. Therefore, both variables were used for the further analysis of the regional distribution of rural/remote regions.

Human resources of health were investigated by looking at the local distribution of professional groups (standardized variables) between remote and other regions. Infrastructural variables for the entire provinces (remote and non-remote regions) showed relevant discrimination or correlative impact (data not shown), while further analysis focused on resources available in remote regions. As expected, the HRH availability in *puskemas* was weak but significantly correlated with GDP per capita (*r*^2^ = 0.16; *p* < 0.05). In contrast, the densities of GP and all physicians were not different.

### Factorial Analysis of the Independent Variables for Healthcare Infrastructure and Population Structure

**Figure 1:**
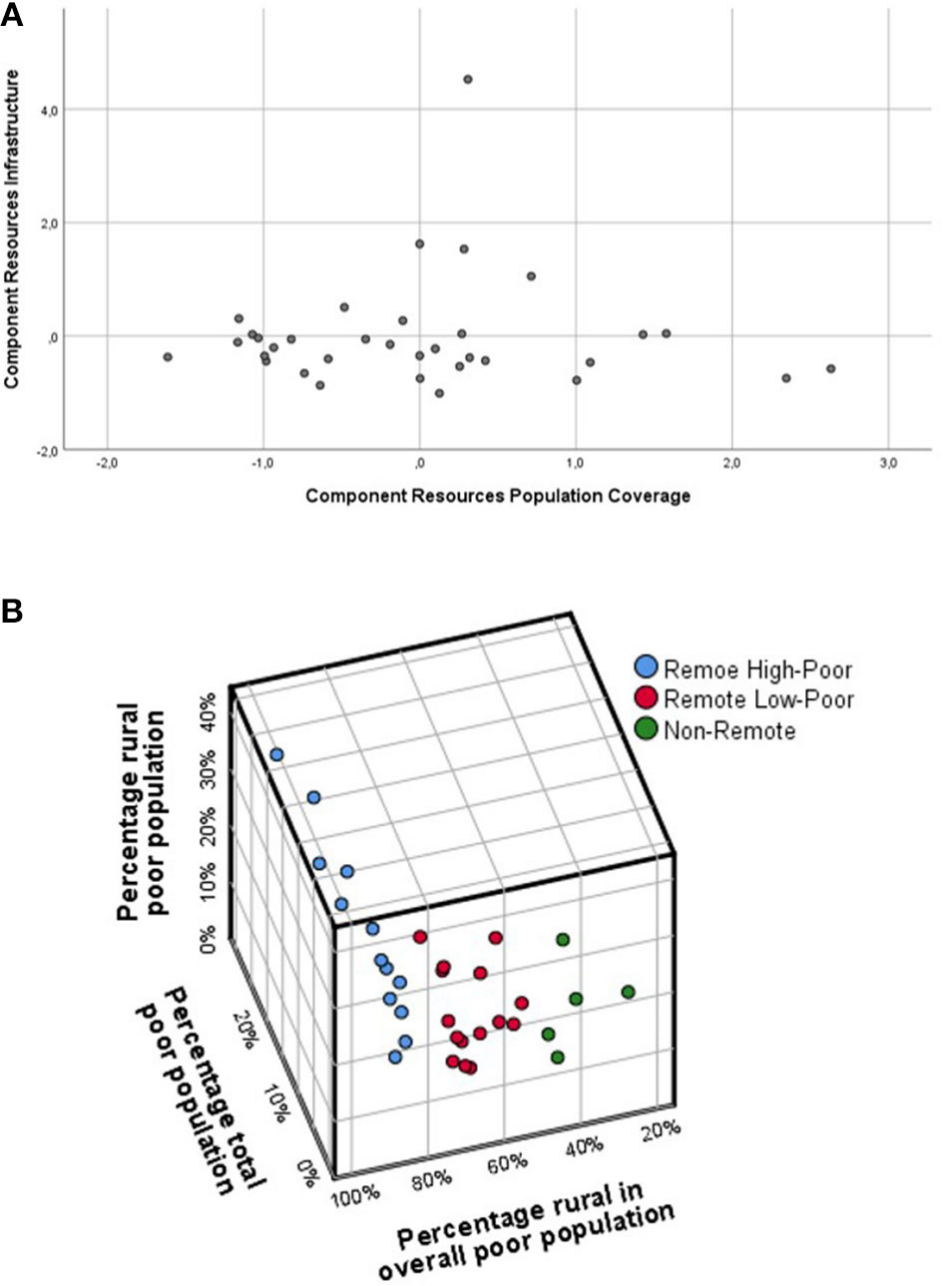
**(A)** Principal component analysis (PCA) for the HRH and infrastructure availability in the provinces. The factorial load for the obtained factors, “population coverage” and “infrastructure,” is shown. DKI Jakarta is included but not considered for subsequent analysis due to its very different characteristics. **(B)** Grouping of provinces according to demographic and economic vulnerability using a ward three-cluster solution; for the avoidance of scaling effects and reduction of parameter variances, they have been further standardized according to $Pstand=\;\frac{Pprov-mean\;\left(Pallprov\right)}{STD\;\left(Pallprov\right)}$. Pstand, standardized parameter per capita in each province; Pprov, parameter in the single province; Pallprov, mean and SD for the parameter of all provinces.

Since the availability of healthcare infrastructure is determined by various infrastructural and human resource variables, a reduction of the number of these variables for further analysis was required. A factorial analysis was used to identify the aggregated factors that can describe this availability. Professional groups (numbers of available healthcare workers) were combined into three HRH groups (GP, medical specialists, nurses/midwives), and overall HRH capacity that were normalized in each province for population (= absolute numbers/1.000 population) and also related to the number of primary care units (= absolute numbers/puskesmas or class A hospital). Seven variables representing healthcare infrastructure (*puskesmas* and hospital beds) and available HRH were correlated (Supplementary Table 3B) and included in the factorial analysis. Two factors explaining >79% of the variance were identified, namely, “population coverage” and “healthcare infrastructure” (Figure 1A). Both factors were used for all subsequent evaluations as descriptions of healthcare resources.

### Clustering of Provinces Regarding Healthcare Infrastructure

To discriminate and group the provinces regarding their demographic characteristics, which were expected to be relevant as determinants for healthcare coverage, a canonical cluster analysis was performed based on the previously discussed population descriptors and infrastructure variables. Different clustering approaches highlighted that the province of Jakarta (DKI) was characterized by various specialties, such as lack of a remote population and different GDP and healthcare structure, mostly resulting in a single-province cluster. Therefore, this province was separated into various analytical approaches. Based on the remaining 33 provinces, the obtained three-cluster solutions (ward procedure) provided the best results (Figure 1B):

- Remote High Poor: the remote region with a high percentage of people in the poor population (*N* = 13).
- Remote Low Poor: the remote region with a low percentage of people in the poor population (*N* = 15).
- Non-Remote: the non-remote region with a low percentage of people in the poor population (*N* = 5).

The subsequent discriminant analysis (Supplementary Tables 4A–C) confirmed that the three input variables significantly (Wilks'-Lambda: *p* < 0.001) discriminated the obtained three clusters that were used for further evaluation (Supplementary Table 5).

**Figure 2:**
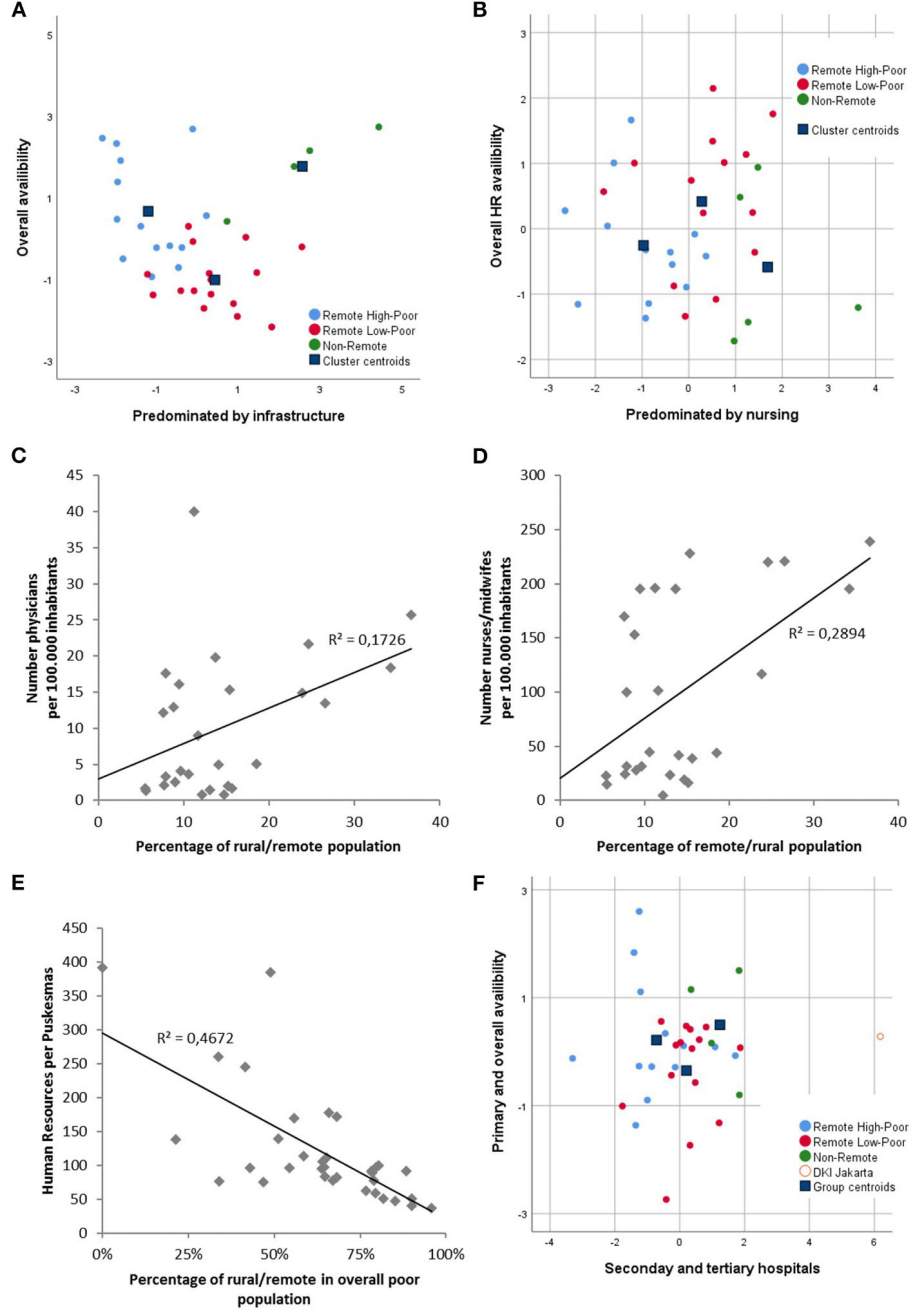
Relationship between healthcare resources and province clusters. Canonical discrimination functions are obtained by infrastructure resource variables for **(A)** the overall healthcare infrastructure and **(B)** HRH. Weak correlations between the percentage of remote populations in provinces and the number of available **(C)** physicians and **(D)** nurses/midwives in remote regions. **(E)** Average HRH per *puskesmas* is related to the percentage of the remote population in the overall poor population. **(F)** Weak differentiation of canonical discrimination functions using hospital bed availability.

In a similar approach, HRH were investigated regarding their distribution between the obtained clusters of provinces. If infrastructure (human resource density, hospital beds, # of *puskesmas*) was included as a characterizing variable for these clusters, high discriminative power was obtained. The canonical discrimination enabled an >82% correct classification by the two obtained discrimination functions, pointing to the fact that healthcare infrastructure differed between provinces with different demographic characteristics. (*p* < 0.005; Figure 2A) Misclassification was solely attributed to the differences between the two clusters with remote regions (Supplementary Figure 1). The standardized densities or population-based availabilities (per 100,000) of nurses/midwivses and overall HRH per *puskesmas* showed significant differences between the clusters (*p* < 0.01), with the lowest values being found in the remote high-poor cluster and the highest densities in the non-remote cluster. Interestingly, if only overall HRH available in remote regions was used for discrimination: differences between the clusters were not significant (data not shown).

### Availability of Resources in Remote and Poor Regions

In the next step, we analyzed whether the province clusters of the provinces could be discriminated based on HRH and infrastructure availability. The population-based availabilities (per 100,000) of HRH in remote regions was very highly correlated between GP, nursing, and all HRH (*r*^2^ > 0.94; *p* < 0.001). Based on these variables, the discrimination of province clusters was weak (Figure 2B), which was mainly supported by the univariate correlation of the proportions of remote regions and the percentages of people in the poor population with HRH variables in these regions (Figures 2C,D). However, by looking at the absolute numbers of the remote population, their comparison with the HRH in remote regions provided a slightly different picture. Large SDs were seen in all groups. Therefore, significant differences were not always found. In general, both GP and nurse/midwife availability were significantly less in remote regions compared to all the provinces (*p* < 0.005), but differences between provinces with high and low impacts of the poor population were not found (Table 1). However, if *puskesmas* as outpatient PHC structures were analyzed regarding their HRH availability and compared with the percentage of the remote population, a significant inverse correlation was found. The percentage of the remote population (*p* < 0.001), the percentage of the remote poor population related to the total population (*p* < 0.05), and the percentage of the remote population in the overall poor population (*p* < 0.001) were significantly correlated with HRH per *puskesmas* (physicians, nurses/midwives, and overall) (Figures 2C–E). Furthermore, low HRH were identified in the remote high-poor cluster (68.9 ± 23.1), intermediate in the remote low-poor cluster (132.4 ± 78.1), and high in the non-remote cluster (163.3 ± 84.9) (Table 1).

**Table 1:** Human resources for healthcare (HRH) in entire provinces and remote regions, given as HRH per 100,000 population and HRH density (covered population per HRH) for general practitioners (GP) and nurses/midwives.

		**HRH per 100,000 population**				**HRH density**			
**Demographic cluster**		**GP**		**Nurses & Midwifes**		**GP**		**Nurses & Midwifes**	
		**Remote regions**	**Province**	**Remote regions**	**Province**	**Remote regions**	**Province**	**Remote regions**	**Province**
Remote High Poor	Mean	11.2**	16.7	201.3*	296.4	29015.6	6588.6	1721.6	359.8*
	±	±	±	±	±	±	±	±	±
	SD	7.5	5.3	128.8	74.6	46334.9	2147.8	3239.1	100
	N	13	13	13	13	13	13	13	13
Remote Low Poor	Mean	7.6**	25	127.7**	298.5	35934	4665.7	1686.5	364.2*
	±	±	±	±	±	±	±	±	±
	SD	8.39	10.5	149.2	92.1	28530.5	1.903.4	1.115.9	105.7
	N	12	15	12	15	12	15	12	15
Non-Remote	Mean	83.9	25.9	917.8	207	12359.2	6653.7	640.7	653.2
	±	±	±	±	±	±	±	±	±
	SD	112.8	19.3	1182.4	107.8	16614.4	5357.2	825.4	412.5
	N	2	5	2	5	2	5	2	5

*Since the clusters “remote high poor” and “remote low poor” were not considered different, significance was given for their comparison to the cluster “non-remote”*

(* *p < 0.01*;

** *p < 0.005)*.

The availability of hospital beds per population was also investigated, including the different classes of hospitals with their related healthcare delivery functions. Although large differences were observed between the provinces (0.77–2.33 beds per 1,000 population), this was only very weakly related to the percentages of the remote and poor population (Figure 2F). If class A and B hospitals were considered as separate primary care access points, the slight correlation was completely lost (data not shown).

### Usage of Healthcare Structures

**Figure 3:**
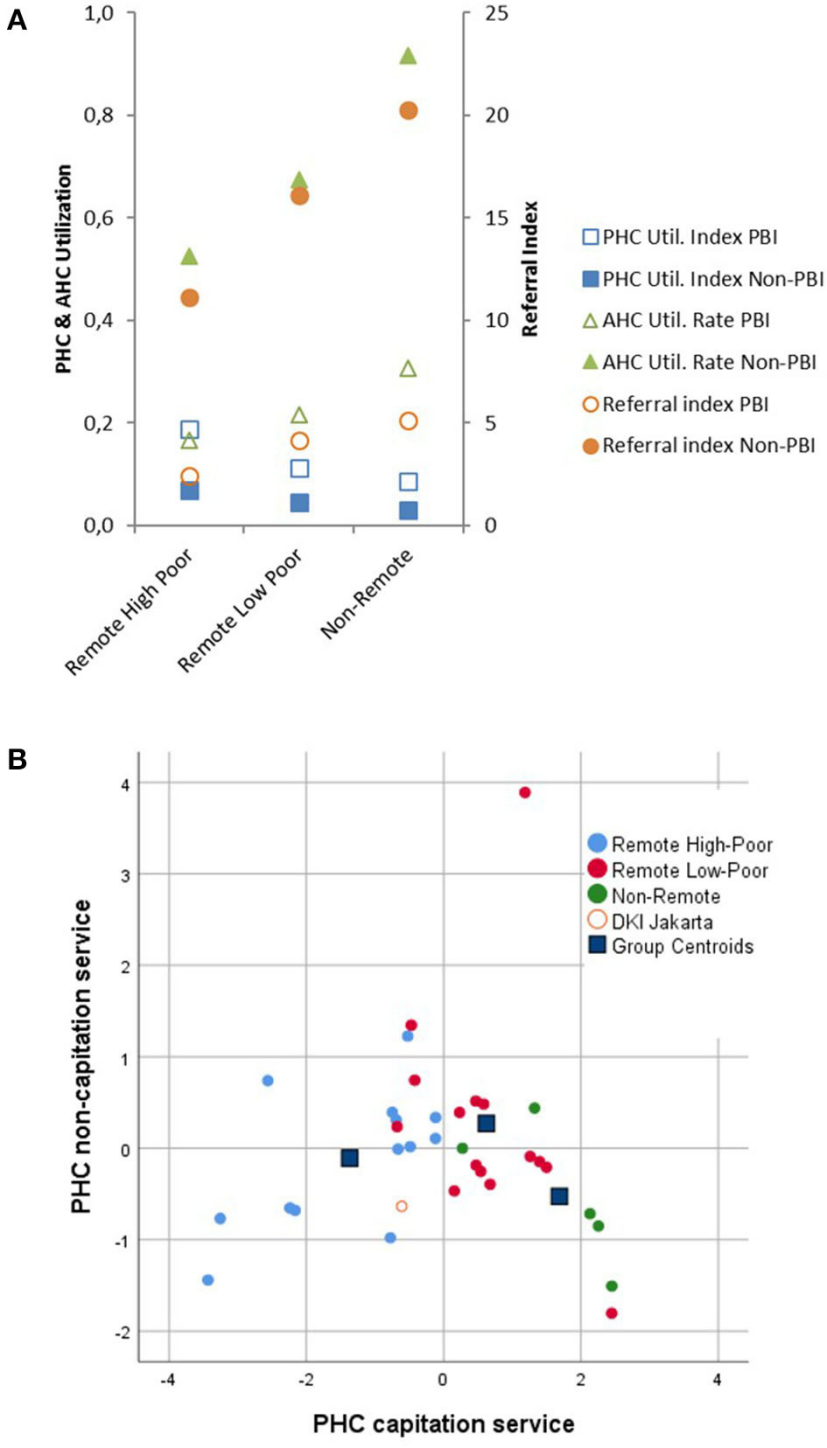
Healthcare services for PBI (□) and Non-PBI insurance (■) members. **(A)** PHC Utilization Index (primary care) (■), AHC Utilization Rate (advanced care) (▴) and AHC/PHC Referral Index (•). **(B)** Canonic discrimination functions obtained by capitation and non-capitation PHC primary services.

The usage of PHC structures is of utmost importance for the effectiveness and efficiency of the Indonesian healthcare system. Therefore, the subsequent analyses focused on PHC in relation to AHC usage and their determinants regarding infrastructure availability and demographic characteristics. Overall, the usage of primary care (PHC) was ~2-fold of advanced care usage (AHC) representing 28.2% and 14.4% of JKN members. PHC capitation reimbursement was considered as principal coverage by JKN, while PHC non-capitation services were taken as indicators for actual primary care service. If included in discrimination analysis, the resulting canonic functions (Figures 3A,B) showed significant differences in primary care service between the population clusters, mainly determined by discrimination coefficients related to indices referring to non-PBI participants and the PBI referral index. Interestingly, for the remote high-poor cluster, the highest primary care PHC utilization indices were observed, whereas the non-remote cluster had the lowest index values. For PBI members, this index was significantly higher (*p* < 0.001) than for non-PBI participants (Figures 3A,B). In contrast, if advanced care AHC utilization rates were analyzed, an inverse behavior was observed. Non-PBI members and the non-remote cluster showed significantly higher usage compared with PBI members and other population clusters (*p* < 0.001). This was also supported by the AHC/PHC referral index representing relations between primary and advanced care utilization (Figure 3B). These differences could be found in the univariate and multivariate comparisons (Supplementary Table 6). Non-PBI members utilized advanced care 4- to 5-fold more frequently than PBI members. Referral indices (*r*^2^ = 0.94; *p* < 0.001) for PBI and non-PBI groups and AHC Utilization Rates (*r*^2^ = 0.53; *p* < 0.001) were highly correlated, whereas PHC utilization indices showed only low correlations between the insurance groups (data not shown). As noticed, the infrastructure for DKI Jakarta differed from all other provinces to a large extent throughout the healthcare usage parameters.

Looking at infrastructural factors influencing usage and referral behavior, it was observed that the availability of HRH at primary care was correlated with the referral and utilization indices reflecting advanced care services. In contrast, the numbers of covered population per *puskemas* were significantly related to utilization indices and utilization rates but not with referral indices (Table 2A). Primary and advanced care utilization were inversely related to each other, demonstrating that primary care was less used in regions where *puskesmas* covered larger groups of the population. Interestingly, the differences between PBI and Non-PBI groups and between the population clusters regarding these correlations were not found in the multivariate approach (Supplementary Figure 2). For an overall evaluation of the utilization variance explained by infrastructure and HRH availability, the two obtained factors, “population coverage” and “infrastructure” (see above), were combined in a canonical correlation analysis. This showed that more than 60% of the utilization variances could be explained by province characteristics. However, this was mainly attributed to the advanced care indices (AHC utilization rate, referral index). The canonical correlation coefficients were 0.9 for obtained set 1 and 0.67 for set 2 (Wilks' test: *p* < 0.001 and *p* < 0.005), respectively (Table 2B).

**Table 2A:** Pearson correlation between factors related to *puskesmas* and service indices (+ *p* < 0.05; ^*^p < 0.005; ^**^p < 0.001).

**(A)**	**Number of HRH per Puskesmas (Standardized)**	**Covered Population per Puskesmas (Standardized)**
PHC Utilization Index PBI	−0.202	−0.433^+^
PHC Utilization Index Non-PBI	−0.263	−0.436^+^
Referral index PBI	0.572**	0.227
Referral index Non-PBI	0.545*	0.226
AHC Utilization Rate PBI	0.714**	0.388^+^
AHC Utilization Rate Non-PBI	0.475*	0.637**

**Table 2B:** Standardized numbers were used for the canonic correlation analysis demonstrating multivariate correlation (Wilks' test, *p* < 0.01).

**(B)**		
	**Standardized canonical correlation coefficients**	
	**Canonical function 1**	**Canonical function 2**
**Set 1 variables**		
PHC Utilization Index PBI	0.152	−0.311
PHC Utilization Index Non-PBI	−0.216	0.007
Referral index PBI	0.205	10.954^§^
Referral index Non-PBI	−0.678^§^	−10.456^§^
AHC Utilization Rate PBI	−0.622^§^	−10.390^§^
AHC Utilization Rate Non-PBI	−0.070	10.395^§^
**Set 2 variables**		
Factor Infrastructure	−0.981^§^	−0.196
Factor Population Coverage	−0.196	0.981^§^

§ *Relevant canonical correlation coefficients >0.5 are highlighted*.

The referral Indexes, but not both the utilization indices and rates, were highly correlated with GDP per capita in the PBI (*r*^2^ = 0.55; *p* < 0.001) and non-PBI (*r*^2^ = 0.64; *p* < 0.001) insurance groups. The age structure of the JKN members was independent of most service indices and only slightly correlated with the AHC utilization index in the non-PBI group (Supplementary Figure 3).

## Discussion

To meet the many challenges inherent in actually delivering affordable healthcare to all Indonesians, the country needs to strengthen its capacity for rigorous evaluation and policy learning at the national and local levels. This would also enable the use of deeper technical evidence to guide the implementation of ambitious plans. Given the diverse range of forms of geography and economic statuses across the country, regular comprehensive assessments of disparities in morbidity, mortality, and disability patterns and their causes are needed. The healthcare system must be able to respond to changing demands due to epidemiological shifts and reduce financial barriers as a result of JKN (20–22). This analysis provided the first evaluation for the new Indonesian health insurance system regarding the availability and accessibility of healthcare resources and delivery processes, with a specific focus on the poor and rural/remote population and PHC utilization. It was based on the entire BPJS dataset. Furthermore, this study initiated the provision of analytical evidence for recommendations regarding the healthcare development strategies at the national and provincial levels.

The provinces of the country can be separated into three different clusters, which were well characterized by the extent of the population in rural/remote regions and the percentage of the poor population. This clustering likely enabled healthcare politics to develop strategies for delivery processes that are generally applicable throughout the country. The obtained clusters showed high discriminative power for differentiation regarding demographic/HRH and healthcare infrastructure data.

Access to PHC is known as an important component of healthcare provision and referral management. It has also been described as a key factor for acceptability and accessibility (23). Therefore, this analysis of healthcare infrastructure focused on resources that are of special importance for primary care, including GP and nurses/midwives for HRH and *puskesmas*/hospital beds (especially for classes A and B) for infrastructure. If these factors were combined in multivariate approaches, resource availability for primary care can be made distinct between the province clusters. This cluster discrimination was rather predominated by infrastructure availability (*puskesmas* and hospital beds). In contrast, HRH availability was intensively related to the extent of the remote population in the provinces but not the numbers of the poor population. The first conclusion that can be drawn from this observation is that the development of HRH for primary care availability should intensively focus on remote regions. This could be supported by telemedicine approaches (24).

Primary care usage, as expressed by PHC non-capitation index and obtained primary care discrimination functions, was found to be much higher in the remote clusters. In contrast, access to AHC was higher in the non-PBI and non-remote populations. This suggested that primary care access points are mainly used by the poor population and people living in rural/remote regions, whereas other population groups prefer direct access to advanced care structures. Similar effects on AHC preference during healthcare insurance implementation (25) and the importance of rural healthcare accessibility (26) were observed in India, China (27), and the US (28). The uneven subscription into the BPJS programs between the poor and non-poor populations likely potentiated these effects, since PBI members were more predominant in remote regions. However, it was unexpected that the preference of AHC was evident in all Indonesian provinces and that the usage rates differed to a large extent, specifically by 4- to 5-fold. Thus, these differences in healthcare usage between PBI and non-PBI members throughout the delivery chain and all provinces need to be explained and targeted.

The present analysis revealed that HRH availability in primary care seems to be a determinant of referral and utilization indices toward advanced care services. Since the numbers of covered population per *puskemas* were significantly related to utilization indices and utilization rates, but not to referral indices, and considering the discussed importance of remote regions rather than the extent of the poor population, a special focus and priority toward the improvement of primary care infrastructures (in combination with corresponding HRH development) in these regions are strongly supported by the results of this analysis.

It can be hypothesized that anticipated or real differences in the provided quality of care, impaired trust in primary care, and insufficient primary care delivery processes play a role in the behavior of BPJS members. However, BPJS data were not suitable for the evaluation of determinants for PHC acceptance. Indonesia still has other problems that affect the implementation of JKN, such as the historically determined culture of poor people seeking healthcare differing from that of average urban people (29, 30). Overall, HRH availability and sufficient qualification will likely remain a challenge in the upcoming years due to persistent limitations in infrastructure related to economic opportunities (31, 32). Therefore, BPJS development should start implementing a structured review process that, for example, targets the adaptation of benefit packages and a reimbursement system for improved coverage of the entire population. Furthermore, in remote regions, competency-driven utilization appeared to be more dominant than referrals determined by the requirements of effective and efficient healthcare delivery chains. Thus, the insufficient availability and accessibility of medical specialists in remote regions was assumed to be an important driving conflict for utilization behavior and PHC acceptance (29).

Some limitations of the present analysis need to be addressed. The specific structure of the BPJS membership, such as family memberships, and the reimbursement system did not allow the direct analysis of delivery processes. However, the very large numbers of insured members, provided services, and broadly standardized insurance schemes enabled the implementation of various indicators, sufficiently describing the healthcare delivery chain. Similarly, models for the analysis of healthcare access in rural areas based on the analysis of healthcare delivery outcomes have been used in other settings and could identify several potential cofactors (33). Similar approaches were suggested for primary care change management targeting underserved rural populations, such as in the UK (34). Furthermore, the completeness of data must be critically considered. Due to the fact that, for several districts, not all data were fully available, the authors used the provincial level for the analysis, for which sufficient data quality was likely to avoid systematic bias. As for most insurance data sources, it cannot be ruled out that the coding for healthcare delivery and diagnoses from the providers contained mistakes. However, parallel to the insurance roll-out BPJS implemented, a data quality program could likely reduce the related bias to a very low level. As an analytical approach, a stepwise regression analysis would have been another option. However, a cluster analysis was used due to the requirement of a clear distinction between provinces regarding their concluded plans of action.

Summarizing the results of this investigation, the authors primarily recommend intensively focus on the guidance of patient referral as a JKN development policy. The primary usage of first access points (primary care facilities) and a guided referral to advanced care as a second step in the healthcare delivery chain appear to likely have high and short-term impacts on the effectiveness and efficiency of the healthcare system, especially in serving the rural population. Guiding the referral and utilization of primary care by setting the right incentives will be the most challenging task for the healthcare politicians of Indonesia in the near future, who want to ensure the effective and efficient use of BPJS (35). This seems to be especially important in non-remote regions and for non-PBI members. Not surprisingly, and with longer perspectives, the development of PHC infrastructures is required in remote high-poor regions. In contrast, the HRH availability appeared to need priority in remote regions, in combination with telemedicine approaches, for PHC availability and referral guidance. These challenges will require the continuous control and implementation of the corresponding reporting and analytical pathways (36). Thus, these analyses should be based on high-quality pragmatic designs and focus on later-stage implementation outcomes (37). A suggested way of reporting is the usage of validated indicators that describe referral processes and their cofactors (38). In particular, the indicators developed in this study appear to fulfill these requests. The paramount importance of maintaining support for PHC and referral centers in remote regions was fully supported by the provided evidence of this analysis. However, further investigation of acceptance barriers will be required to fully understand the reasons for such and to provide evidence for strategic healthcare development.

## Data Availability Statement

The data analyzed in this study is subject to the following licenses/restrictions; insurance data are protected by legal issues from public availability. Requests to access these datasets should be directed to andi.afdal@bpjs-kesehatan.go.id.

## Ethics Statement

The studies involving human participants were reviewed and approved by Muhammadiyah University (No. 202/EC-KEPK FKIK UMY/Vlll/2020) and the Indonesian National Healthcare Insurance BPJS (No. 5060/I.2/0419). Written informed consent for participation was not required for this study in accordance with the national legislation and the institutional requirements.

## Author Contributions

SW: concept, data analysis, writing, and discussion. JS: concept and results in interpretation. AA: data provision and data quality. SG: data analysis and methodological discussion. ID: concept and data interpretation. JH: concept, data analysis, and writing. AG: concept and data provision. All authors contributed to the article and approved the submitted version.

## Funding

This project was supported by grants from the Center for Research, Publication, and Community Development Muhammadiyah University of Yogyakarta (SW and ID). Data were provided by BPJS Indonesia.

## Conflict of Interest

AA and AG are employed by BPJS National Healthcare Insurance Indonesia. The remaining authors declare that the research was conducted in the absence of any commercial or financial relationships that could be construed as a potential conflict of interest.

## Publisher's Note

All claims expressed in this article are solely those of the authors and do not necessarily represent those of their affiliated organizations, or those of the publisher, the editors and the reviewers. Any product that may be evaluated in this article, or claim that may be made by its manufacturer, is not guaranteed or endorsed by the publisher.
