# Supplementary material for: Availability and Accessibility of Primary Care for the Remote, Rural, and Poor Population of Indonesia
Source: Front Public Health. 2021 Sep 21;9:721886. doi: 10.3389/fpubh.2021.721886 (PMC8491579; doi:10.3389/fpubh.2021.721886)
Supplement: Supplementary file 2 [file Data_Sheet_2.pdf]

Supplementary data and information

Supplement Figure S1: Clusters of poor and 3T populations can be discriminated by building 2 functions that use the parameters a) Standardized Number of All Physicians per 100.000 Population; b) Standardized Number of All Nursing per 100.000 Population; c) Standardized Number of All Healthcare HRH per 100.000 Population; d) Standardized Average Population per Puskesmas; e) Standardized Number Hospital Beds per 1000 Population; and f) Standardized Number of HRH per Puskesmas. 79.4% of the provinces were correctly classified using this discrimination model. If only human resource factors (overall and in 3T areas) or infrastructure factors were use discrimination became worse.

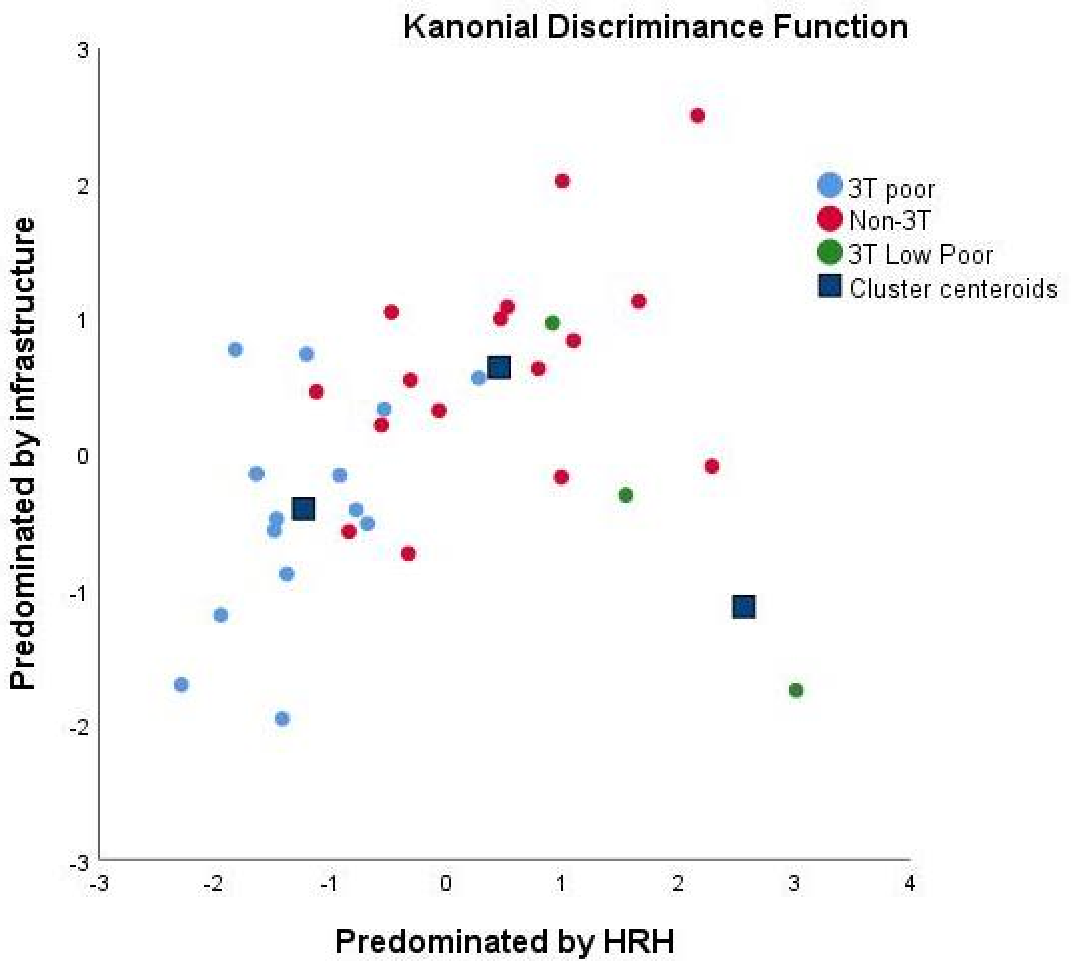

Supplement Figure S2: Referral Index from primary to secondary care. A) PBI, B) Non-PBI

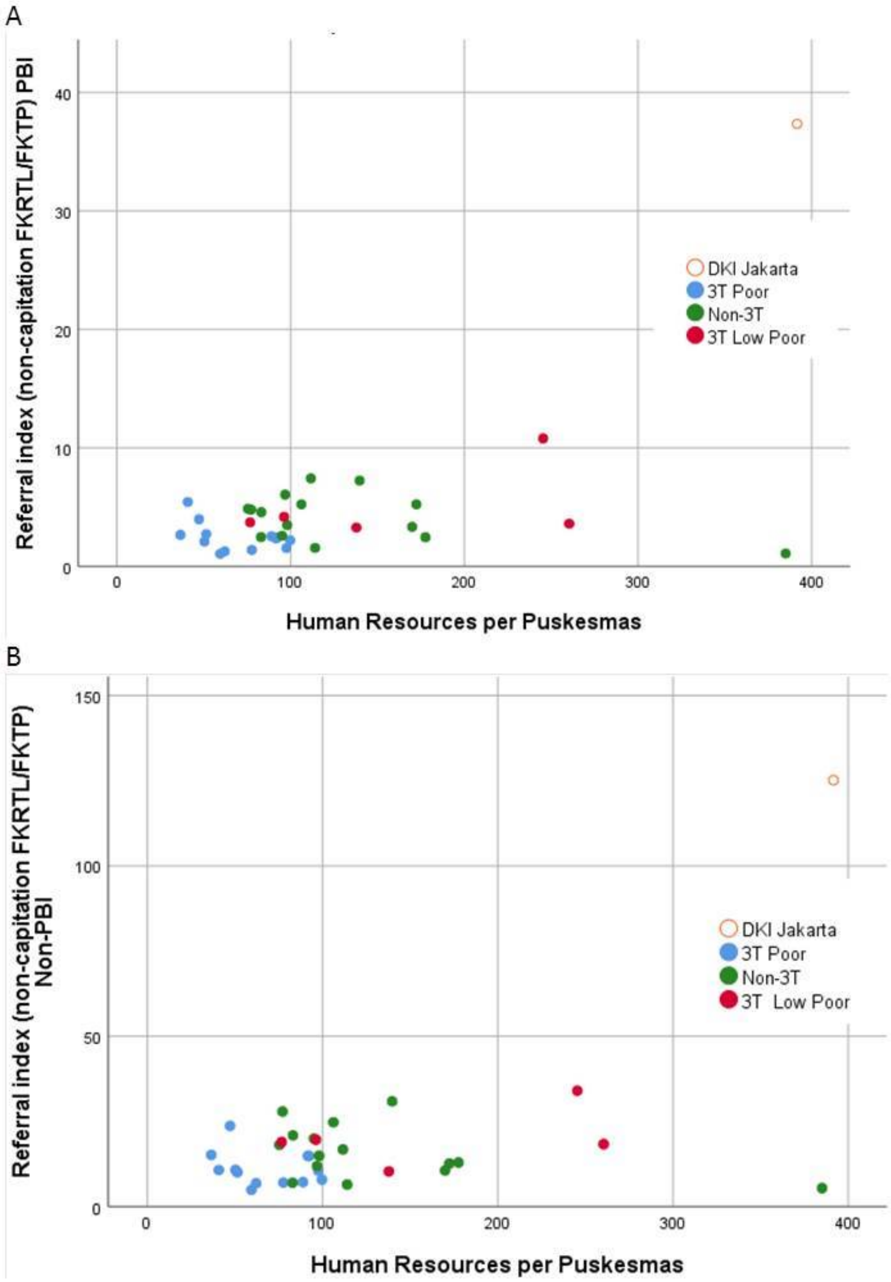

Supplement Figure S3: FKRTL Utilization Rate Non-PBI of non-poor JKN participants in A) seniors and B) adults

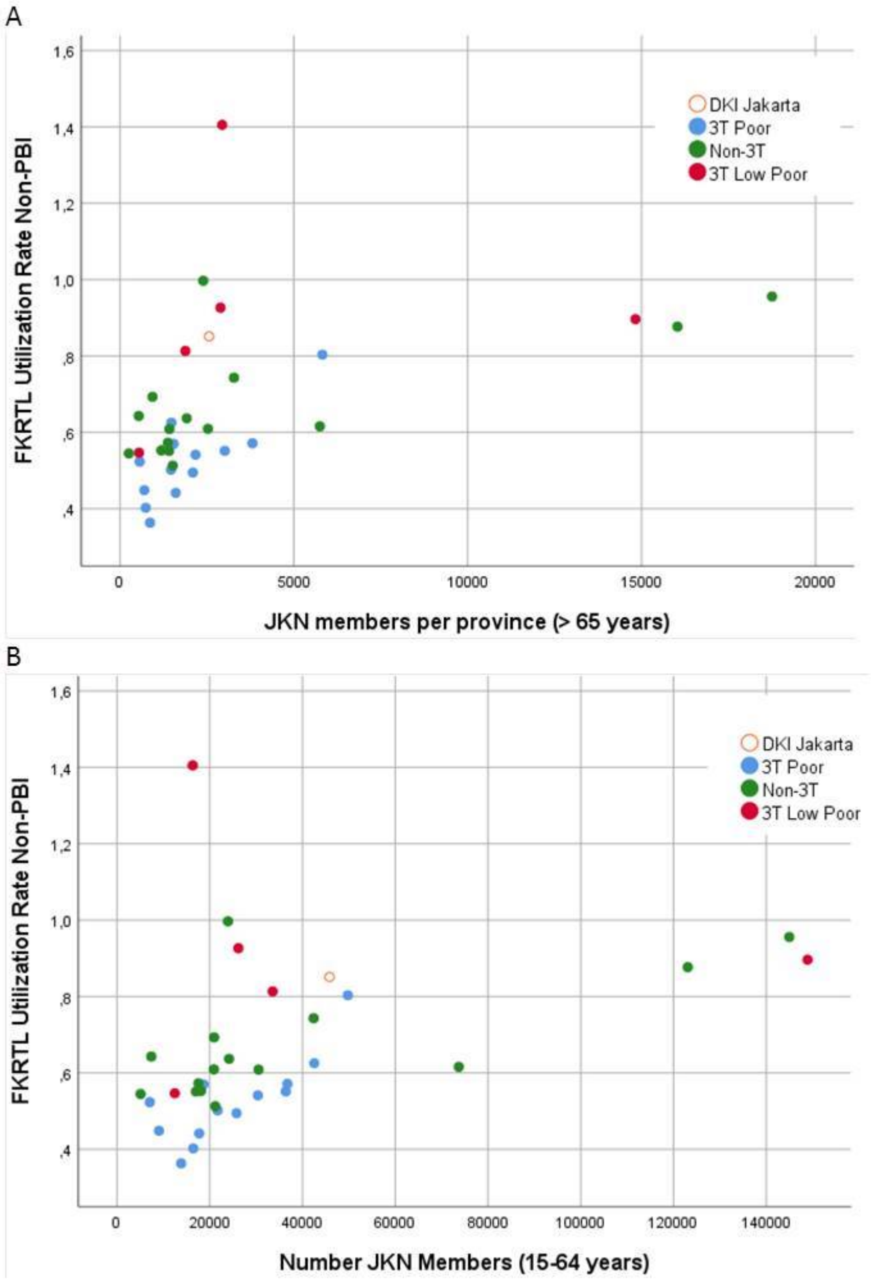

Supplement Table S1: List of used parameters

1	ProvinceName	Province
2	ProvincePDRBCapita	Domestic gross product Per Capita (Ribu Rp)
3	ProvinceGDPCapitaStand	Domestic gross product Per Capita standardized
4	ProvinceDistricts	Number of Districts per Province
5	PopulationTotalNumber	Total population
6	PopulationUrbanNumber	Total urban population
7	PopulationRuralNumber	Total rural population
8	PopulationRuralPercent	Percentage rural population
9	PopulationPoorUrban1000	Number of urban poor population *1000
10	PopulationPoorUrbanNumber	Number of urban poor population absolute
11	PopulationPoorUrbanPercent	Percentage of urban poor population related to total population
12	PopulationPoorRural1000	Number of rural poor population *1000
13	PopulationPoorRuralNumber	Number of rural poor population absolute
14	PopulationPoorRuralPercent	Percentage of rural poor population related to total population
15	PopulationPoorTotal1000	Number of total poor population *1000
16	PopulationPoorTotalNumber	Number of total poor population absolute
17	PopulationNonPoorTotalNumber	Number of total low poor population absolute
18	PopulationNonPoorTotalPercent	Percentage of total low poor population related to total
19	PopulationPoorTotalPercent	Percentage of total poor population related to total population
20	PopulationPoorTotalRuralPercent	Percentage of rural in overall poor population
21	BPJSParicipantsTotal	Total of JKN Participants
22	BPJSPartNonPBI_PBPU	Non PBI PBPU (Non-Wage recipients workers)
23	BPJSPartNonPBI_PPU	Non PBI PPU (Wage recipients workers)
24	BPJSPartNonPBI_BP	Non PBI BP (Not workers)
25	BPJSPartPBI_APBN	PBI APBN (National support)
26	BPJSPartPBI_APBD	PBI Pemda (District support)
27	BPJSPartPoor	Sum poor participants (PBI APBN+APBD)
28	BPJSPartNonPoor	Sum low poor participants (PBPU+PPU+BP)
29	BPJSPartGenderMale	Male
30	BPJSPartGenderFemale	Female
31	BPJSPartAgeYoung	Young Age (<15 years)
32	BPJSPartAgeAdult	Productive Age (15-64 years)
33	BPJSPartAgeSenior	Non Productive Age (> 65 years)
34	BPJSPartMarriageSingle	Single
35	BPJSPartMarriageMarriage	Marriage
36	BPJSPartMarriageDivorce	Divorce
37	BPJSPartMarriageUndefinde	Undefined
38	BPJSPartUsagePrimaryNumber	JKN Participants Visit FKTP
39	BPJSPartUsagePrimaryPercent	Percentage of JKN Participants Visit FKTP (usage rate)
40	BPJSPartUsageSecondaryNumber	JKN Participants Visit FKRTL
41	BPJSPartUsageScondaryPercent	Percentage of JKN Participants Visit FKRTL (usage rate)
42	HospitalClassATotal	Number of Class A hospitals
43	HospitalClassABeds	Number of beds in ClassA hospitals
44	HospitalClassAPercent	Percentage of ClassA hospitals related to all hospitals
45	HospitalClassABedsPop	Number of ClassA hospital beds per 1000 population
46	HospitalClassBTotal	Number of Class B hospitals
47	HospitalClassBBeds	Number of beds in ClassB hospitals
48	HospitalClassBPercent	Percentage of ClassB hospitals related to all hospitals

49	HospitalClassBBedsPop	Number of ClassB hospital beds per 1000 population
50	HospitalClassCTotal	Number of ClassC hospitals
51	HospitalClassCBeds	Number of beds in ClassC hospitals
52	HospitalClassCPercent	Percentage of ClassC hospitals related to all hospitals
53	HospitalClassCBedsPop	Number of ClassC hospital beds per 1000 population
54	HospitalClassDTotal	Number of ClassD (+ D pramata)hospitals
55	HospitalClassDBeds	Number of beds in ClassD (+ D pramata) hospitals
56	HospitalClassDPercent	Percentage of ClassD (+ D pramata) hospitals related to all
57	HospitalClassDBedsPop	Number of ClassD (+ D pramata) hospital beds per 1000
58	HospitalClassNdTotal	Number of Class Not Decided hospitals
59	HospitalClassNdBeds	Number of beds in Class Not Decided hospitals
60	HospitalClassNdPercent	Percentage of Class Not Decided hospitals related to all
61	HospitalClassNdBedsPop	Number of Class not decided hospital beds per 1000 population
62	HospitalTotalTotal	Number of total hospitals
63	HospitalTotalBeds	Number of beds in total hospitals
64	HospitalTotalBedsPop	Number of total hospital beds per 1000 population
65	OutpatientPuskemasNumber	Number of puskesmas per province
66	OutpatientPuskemasHRTotal	Total of Human Resources in all Puskesmas
67	OutpatientPuskemasRatioDistrict	Ratio of puskesmas per district
68	OutpatientPuskemasHRRatio	Human Resources per Puskesmas
69	OutpatientPuskesmasPopCoverage	Average number of population per puskesmas
70	OutpatientGPindependHR	Total of Independent General Practitioners
71	OutpatientFacilititesBPJSContracts	First-level health facilities in collaboration with BPJS
72	OutpatientFacilitiesTotal	Total of puskesmas & independent GP
73	BPJSPartHospitalNursingClass1	Nursing Class I at hospital
74	BPJSPartHospitalNursingClass2	Nursing Class II at hospital
75	BPJSPartHospitalNursingClass3	Nursing Class III at hospital
76	BPJSPartHospitalNursingClassMissing	Nursing Class Missing at hospital
77	BPJSPartRegisterPuskesmas	Total of BPJS participants registered at primary care types
78	BPJSPartRegisterPratamaClinic	Total of BPJS participants registered at primary care types
79	BPJSPartRegisterGenPractice	Total of BPJS participants registered at primary care types
80	RuralHRSpecialistDoctorTotal	HRH in remote regions (Specialist Doctors)
81	RuralHRGP	HRH in remote regions (GP)
82	RuralHRSpecialistDentist	HRH in remote regions (Specialist Dentist)
83	RuralHRDentist	HRH in remote regions (Dentist)
84	RuralHRPsychol	HRH in remote regions (Psychologist)
85	RuralHRPhysiciansTotal	HRH in remote regions (Doctors+GP+Dent+SpecDent+Psychol)
86	RuralHRNurse	HRH in remote regions (Nursing)
87	RuralHRMidwife	HRH in remote regions (Midwife)
88	RuralHRNursingAll	HRH in remote regions (Nurse+Midwife)
89	RuralHRPharma	HRH in remote regions (Pharmaceutical Workers)
90	RuralHRPubliHealth	HRH in remote regions (Public Health)
91	RuralHREnvironment	HRH in remote regions (Environmental Health Workers)
92	RuralHRNutritionist	HRH in remote regions (Nutritionist)
93	RuralHRPhysical	HRH in remote regions (Physical Absorption Workers)
94	RuralHRMedTech	HRH in remote regions (Medical Technical Workers)
95	RuralHRBiomed	HRH in remote regions (Biomedical Engineering Personnel)
96	RuralHRTraditional	HRH in remote regions (Traditional Health Workers)
97	RuralHRHealthSupport	HRH in remote regions (Health Support Workers)
98	RuralHRTotal	HRH in remote regions (Total of Human Resources for Health)

99	RuralHRPopSpecialistDoctorTotal	HRH in remote regions per 100.000 rural population (Specialist
100	RuralHRPopGP	HRH in remote regions per 100.000 rural population (GP)
101	RuralHRPopSpecialistDentist	HRH in remote regions per 100.000 rural population (Specialist
102	RuralHRPopDentist	HRH in remote regions per 100.000 rural population (Dentist)
103	RuralHRPopPsychol	HRH in remote regions per 100.000 rural population
104	RuralHRPopPhysiciansTotal	HRH in remote regions per 100.000 rural population
105	RuralHRPopNurse	HRH in remote regions per 100.000 rural population (Nursing)
106	RuralHRPopMidwife	HRH in remote regions per 100.000 rural population (Midwife)
107	RuralHRPopNursingAll	HRH in remote regions per 100.000 rural population
108	RuralHRPopPharma	HRH in remote regions per 100.000 rural population
109	RuralHRPopPubliHealth	HRH in remote regions per 100.000 rural population (Public
110	RuralHRPopEnvironment	HRH in remote regions per 100.000 rural population
111	RuralHRPopNutritionist	HRH in remote regions per 100.000 rural population
112	RuralHRPopPhysical	HRH in remote regions per 100.000 rural population (Physical
113	RuralHRPopMedTech	HRH in remote regions per 100.000 rural population (Medical
114	RuralHRPopBiomed	HRH in remote regions per 100.000 rural population
115	RuralHRPopTraditional	HRH in remote regions per 100.000 rural population (Traditional
116	RuralHRPopHealthSupport	HRH in remote regions per 100.000 rural population (Health
117	RuralHRPopTotal	HRH in remote regions per 100.000 rural population (Total of
118	RuralHRDensitySpecialistDoctorTotal	HRH density rural population per healthworker (Specialist
119	RuralHRDensityGP	HRH density rural population per healthworker (GP)
120	RuralHRDensitySpecialistDentist	HRH density rural population per healthworker (Specialist
121	RuralHRDensityDentist	HRH density rural population per healthworker (Dentist)
122	RuralHRDensityPsychol	HRH density rural population per healthworker (Psychologist)
123	RuralHRDensityPhysiciansTotal	HRH density rural population per healthworker
124	RuralHRDensityNurse	HRH density rural population per healthworker(Nursing)
125	RuralHRDensityMidwife	HRH density rural population per healthworker(Midwife)
126	RuralHRDensityNursingAll	HRH density rural population per healthworker
127	RuralHRDensityPharma	HRH density rural population per healthworker(Pharmaceutical
128	RuralHRDensityPubliHealth	HRH density rural population per healthworker(Public Health)
129	RuralHRDensityEnvironment	HRH density rural population per healthworker(Environmental
130	RuralHRDensityNutritionist	HRH density rural population per healthworker(Nutritionist)
131	RuralHRDensityPhysical	HRH density rural population per healthworker(Physical
132	RuralHRDensityMedTech	HRH density rural population per healthworker(Medical
133	RuralHRDensityBiomed	HRH density rural population per healthworker(Biomedical
134	RuralHRDensityTraditional	HRH density rural population per healthworker(Traditional
135	RuralHRDensityHealthSupport	HRH density rural population per healthworker(Health Support
136	RuralHRDensityTotal	HRH density rural population per healthworker(Total of Human
137	RuralHRPercentSpecialistDoctorTotal	HRH Percentage in rural of all available (Specialist Doctors)
138	RuralHRPercentGP	HRH Percentage in rural of all available (GP)
139	RuralHRPercentSpecialistDentist	HRH Percentage in rural of all available (Specialist Dentist)
140	RuralHRPercentDentist	HRH Percentage in rural of all available (Dentist)
141	RuralHRPercentPsychol	HRH Percentage in rural of all available (Psychologist)
142	RuralHRPercentNurse	HRH Percentage in rural of all available (Nursing)
143	RuralHRPercentMidwife	HRH Percentage in rural of all available (Midwife)
144	RuralHRPercentPharma	HRH Percentage in rural of all available (Pharmaceutical
145	RuralHRPercentPubliHealth	HRH Percentage in rural of all available (Public Health)
146	RuralHRPercentEnvironment	HRH Percentage in rural of all available (Environmental Health
147	RuralHRPercentNutritionist	HRH Percentage in rural of all available (Nutritionist)
148	RuralHRPercentPhysical	HRH Percentage in rural of all available (Physical Absorption

149	RuralHRPercentMedTech	HRH Percentage in rural of all available (Medical Technical
150	RuralHRPercentBiomed	HRH Percentage in rural of all available (Biomedical Engineering
151	RuralHRPercentTraditional	HRH Percentage in rural of all available (Traditional Health
152	RuralHRPercentHealthSupport	HRH Percentage in rural of all available (Health Support)
153	TotalHRNumberSpecialistDoctorTotal	HRH Total available (Specialist Doctors)
154	TotalHRNumberGP	HRH Total available (GP)
155	TotalHRNumberSpecialistDentist	HRH Total available (Specialist Dentist)
156	TotalHRNumberDentist	HRH Total available (Dentist)
157	TotalHRNumberPsychol	HRH Total available (Psychologist)
158	TotalHRNumberPhysicians	HRH Total available (Doctors+GP+Dent+SpecDent+Psychol)
159	TotalHRNumberNurse	HRH Total available (Nursing)
160	TotalHRNumberMidwife	HRH Total available (Midwife)
161	TotalHRNumberNursingAll	HRH Total available (Nurse+Midwife)
162	TotalHRNumberPharma	HRH Total available (Pharmaceutical Workers)
163	TotalHRNumberPubliHealth	HRH Total available (Public Health)
164	TotalHRNumberEnvironment	HRH Total available (Environmental Health Workers)
165	TotalHRNumberNutritionist	HRH Total available (Nutritionist)
166	TotalHRNumberPhysical	HRH Total available (Physical Absorption Workers)
167	TotalHRNumberMedTech	HRH Total available (Medical Technical Workers)
168	TotalHRNumberBiomed	HRH Total available (Biomedical Engineering Personnel)
169	TotalHRNumberTraditional	HRH Total available (Traditional Health Workers)
170	TotalHRNumberHealthSupport	HRH Total available (Health Support)
171	TotalHRPopSpecialistDoctorTotal	HRH Total available per 100.000 Population (Specialist Doctors)
172	TotalHRPopGP	HRH Total available per 100.000 Population (GP)
173	TotalHRPopSpecialistDentist	HRH Total available per 100.000 Population (Specialist Dentist)
174	TotalHRPopDentist	HRH Total available per 100.000 Population (Dentist)
175	TotalHRPopPsychol	HRH Total available per 100.000 Population (Psychologist)
176	TotalHRPopPhysicians	HRH Total available per 100.000 Population
177	TotalHRPopNurse	HRH Total available per 100.000 Population (Nursing)
178	TotalHRPopMidwife	HRH Total available per 100.000 Population (Midwife)
179	TotalHRPopNursingAll	HRH Total available per 100.000 Population (Nurse+Midwife)
180	TotalHRPopPharma	HRH Total available per 100.000 Population (Pharmaceutical
181	TotalHRPopPubliHealth	HRH Total available per 100.000 Population (Public Health)
182	TotalHRPopEnvironment	HRH Total available per 100.000 Population (Environmental
183	TotalHRPopNutritionist	HRH Total available per 100.000 Population (Nutritionist)
184	TotalHRPopPhysical	HRH Total available per 100.000 Population (Physical
185	TotalHRPopMedTech	HRH Total available per 100.000 Population (Medical Technical
186	TotalHRPopBiomed	HRH Total available per 100.000 Population (Biomedical
187	TotalHRPopTraditional	HRH Total available per 100.000 Population (Traditional Health
188	TotalHRPopHealthSupport	HRH Total available per 100.000 Population (Health Support)
189	TotalHRDensitySpecialistDoctorTotal	HRH Total density population per healthworker (Specialist
190	TotalHRDensityGP	HRH Total density population per healthworker (GP)
191	TotalHRDensitySpecialistDentist	HRH Total density population per healthworker (Specialist
192	TotalHRDensityDentist	HRH Total density population per healthworker (Dentist)
193	TotalHRDensityPsychol	HRH Total density population per healthworker (Psychologist)
194	TotalHRDensityPhysicians	HRH Total density population per healthworker
195	TotalHRDensityNurse	HRH Total density population per healthworker (Nursing)
196	TotalHRDensityMidwife	HRH Total density population per healthworker (Midwife)
197	TotalHRDensityNursingAll	HRH Total density population per healthworker
198	TotalHRDensityPharma	HRH Total density population per healthworker (Pharmaceutical

199	TotalHRDensityPubliHealth	HRH Total density population per healthworker (Public Health)
200	TotalHRDensityEnvironment	HRH Total density population per healthworker (Environmental
201	TotalHRDensityNutritionist	HRH Total density population per healthworker (Nutritionist)
202	TotalHRDensityPhysical	HRH Total density population per healthworker (Physical
203	TotalHRDensityMedTech	HRH Total density population per healthworker (Medical
204	TotalHRDensityBiomed	HRH Total density population per healthworker (Biomedical
205	TotalHRDensityTraditional	HRH Total density population per healthworker (Traditional
206	TotalHRDensityHealthSupport	HRH Total density population per healthworker (Health
207	ServiceFKTP_CapBPTotal	FKTP capitation service BP
208	ServiceFKTP_CapBPPercent	FKTP capitation service Percentage of all FKTP BP
209	ServiceFKTP_CapPBI_APBNTotal	FKTP capitation service PBI APBN
210	ServiceFKTP_CapPBI_APBNPercent	FKTP capitation service Percentage of all FKTP PBI APBN
211	ServiceFKTP_CapPBI_APBDTotal	FKTP capitation service PBI APBD
212	ServiceFKTP_CapPBI_APBDPercent	FKTP capitation service Percentage of all FKTP PBI APBD
213	ServiceFKTP_Cap_Poor_Total	FKTP capitation service PBI APBN+APBD
214	ServiceFKTP_CapPBPUTotal	FKTP capitation service PBPU
215	ServiceFKTP_CapPBPUPercent	FKTP capitation service Percentage of all FKTP PBPU
216	ServiceFKTP_CapPPUTotal	FKTP capitation service PPU
217	ServiceFKTP_CapPPUPercent	FKTP capitation service Percentage of all FKTP PPU
218	ServiceFKTP_CapMissingTotal	FKTP capitation service Missing
219	ServiceFKTP_CapMissingPercent	FKTP capitation service Percentage of all FKTP Missing
220	ServiceFKTP_Cap_NonPoor_Total	FKTP capitation service BP & PBPU & PPU
221	ServiceFKTP_CapTotal	FKTP capitation service Total
222	ServiceFKTP_NonCapBPTotal	FKTP non capitation service BP
223	ServiceFKTP_NonCapBPPercent	FKTP non capitation service Percentage of all FKTP non BP
224	ServiceFKTP_NonCapPBI_APBNTotal	FKTP non capitation service PBI APBN
225	ServiceFKTP_NonCapPBI_APBNPercent	FKTP non capitation service Percentage of all FKTP non PBI
226	ServiceFKTP_NonCapPBI_APBDTotal	FKTP non capitation service PBI APBD
227	ServiceFKTP_NonCapPBI_APBDPercent	FKTP non capitation service Percentage of all FKTP non PBI
228	ServiceFKTP_NonCap_Poor_Total	FKTP non capitation service PBI APBN+APBD
229	ServiceFKTP_NonCapPBPUTotal	FKTP non capitation service PBPU
230	ServiceFKTP_NonCapPBPUPercent	FKTP non capitation service Percentage of all FKTP non PBPU
231	ServiceFKTP_NonCapPPUTotal	FKTP non capitation service PPU
232	ServiceFKTP_NonCapPPUPercent	FKTP non capitation service Percentage of all FKTP non PPU
233	ServiceFKTP_NonCapMissingTotal	FKTP non capitation service Missing
234	ServiceFKTP_NonCapMissingPercent	FKTP non capitation service Percentage of all FKTP non Missing
235	ServiceFKTP_NonCap_NonPoor_Total	FKTP non capitation service BP & PBPU & PPU
236	ServiceFKTP_NonCapTotal	FKTP non capitation service Total
237	ServiceFKRTL_BPTotal	FKRTL non capitation service BP
238	ServiceFKRTL_BPPercent	FKRTL non capitation service Percentage of all FKRTL non BP
239	ServiceFKRTL_PBI_APBNTotal	FKRTL non capitation service PBI APBN
240	ServiceFKRTL_PBI_APBNPercent	FKRTL non capitation service Percentage of all FKRTL non PBI
241	ServiceFKRTL_PBI_APBDTotal	FKRTL non capitation service PBI APBD
242	ServiceFKRTL_PBI_APBDPercent	FKRTL non capitation service Percentage of all FKRTL non PBI
243	ServiceFKRTL_Poor_Total	FKRTL non capitation service PBI APBN+APBD
244	ServiceFKRTL_PBPUTotal	FKRTL non capitation service PBPU
245	ServiceFKRTL_PBPUPercent	FKRTL non capitation service Percentage of all FKRTL non
246	ServiceFKRTL_PPUTotal	FKRTL non capitation service PPU
247	ServiceFKRTL_PPUPercent	FKRTL non capitation service Percentage of all FKRTL non PPU
248	ServiceFKRTL_MissingTotal	FKRTL non capitation service Missing

249	ServiceFKRTL_MissingPercent	FKRTL non capitation service Percentage of all FKRTL non
250	ServiceFKRTL_NonPoor_Total	FKRTL non capitation service BP & PBPU & PPU
251	ServiceFKRTL_Total	FKRTL non capitation service Total
252	ServiceAllForms_BP	All BPJS service forms BP
253	ServiceAllForms_PBI_APBN	All BPJS service forms PBI APBN
254	ServiceAllForms_PBI_APBD	All BPJS service forms PBI APBD
255	ServiceAllForms_Poor	All BPJS service forms PBI APBN & PBI APBD
256	ServiceAllForms_PBPU	All BPJS service forms PBPU
257	ServiceAllForms_PPU	All BPJS service forms PPU
258	ServiceAllForms_NonPoor	All BPJS service forms BP+PBPU+PPU
259	ServiceAllForms_Total	All BPJS service forms TOTAL
260	ServicePoorPop	Service poor (ABPN+ABPD) for poor population
261	ServiceNonPoorPop	Service low poor (BP+PBPU+PPU) for low poor population
262	ServiceNonAllPop	Service All for Total population
263	ServiceReferalFKRTL_FKTPPoor	Referal index of primary to secondary care (non-capitation
264	ServiceReferalFKRTL_FKTPNonPoor	Referal index of primary to secondary care (non-capitation
265	ServiceReferalFKRTL_FKTPAll	Referal index of primary to secondary care (non-capitation
266	ServiceUsageFKRTL_PartPoor	Usage rate FKRTL of all participants poor
267	ServiceUsageFKRTL_PartNonPoor	Usage rate FKRTL of all participants low poor
268	ServiceUsageFKRTL_PartAll	Usage rate FKRTL of all participants All
269	ServiceAllNonCapitation_BP	All Non-Capitation service (FKTP+FKRTL) BP
270	ServiceAllNonCapitation_PBI_APBN	All Non-Capitation service (FKTP+FKRTL) PBI APBN
271	ServiceAllNonCapitation_PBI_APBD	All Non-Capitation service (FKTP+FKRTL) PBI APBD
272	ServiceAllNonCapitation_Poor	All Non-Capitation service (FKTP+FKRTL) PBI APBN & PBI APBD
273	ServiceAllNonCapitation_PBPU	All Non-Capitation service (FKTP+FKRTL) PBPU
274	ServiceAllNonCapitation_PPU	All Non-Capitation service (FKTP+FKRTL) PPU
275	ServiceAllNonCapitation_NonPoor	All Non-Capitation service (FKTP+FKRTL) BP+PBPU+PPU
276	ServiceAllNonCapitation_Total	All Non-Capitation service (FKTP+FKRTL) TOTAL
277	ServicePoorPopJKN	Service poor (ABPN+ABPD) for poor population JKN members
278	ServiceNonPoorPopJKN	Service low poor (BP+PBPU+PPU) for low poor population JKN
279	ServiceNonAllPopJKN	Service All for Total population JKN members
280	NonCapServicePoorPopJKN	Service poor (ABPN+ABPD) for poor population JKN members
281	NonCapServiceNonPoorPopJKN	Service low poor (BP+PBPU+PPU) for low poor population JKN
282	NonCapServiceNonAllPopJKN	Service All for Total population JKN members

Table S2: Distribution of the Number (Mio) and Percentage (%) of BPJS Healthcare Insurance Participants by Type of Membership, 2014-2017.

Type of Membership	2014	2015	2016	2017*
Recipient of PBI APBN	86.400 (64.8%)	87.829 (56.0%)	91.099 (53.0%)	91.998 (51.6%)
Recipients of PBI APBD	8.767 (6.6%)	11,171 (7.1%)	15.415 (9.0%)	17.110 (9.6%)
Wage Recipient Worker (PPU)	24.327 (18.2%)	37.863 (24.1%)	41.027 (23.9%)	42.178 (23.6%)
Workers Non-Recipients (PBPU)	9.053 (6.8%)	14.962 (9.5%)	19.337 (11.2%)	22.078 (12.4%)
Not worker (BP)	4.876 (3.7%)	4.967 (3.2%)	5.061 (2.9%)	5.020 (2.8%)
**Total**	**133.424**	**156.790**	**171.939**	**178.384**

Table S3: Initial Pearson correlation of standardized raw data

a) Pearson correlation for variables representing healthcare infrastructure (n=34). Significant at **.P<0,01 and * P<0,05 (2-sided).

	Standardized Number Hospital Beds per 1000 Population	Standardized Number of HRH per Puskesmas	Standardized Ratio of Puskesmas per District	Standardized Number of All Physicians per 100.000 Population	Standardized Number of All Nursing per 100.000 Population	Standardized Number of All Healthcare HRH per 100.000 Population
Standardized Average Population per Puskesmas	-,140	,495**	,291	,225	-,538**	-,310
Standardized Number Hospital Beds per 1000 Population		,297	,477**	,554**	,323	,471**
Standardized Number of HRH per Puskesmas			,630**	,886**	,358*	,623**
Standardized Ratio of Puskesmas per District				,751**	,152	,454**
Standardized Number of All Physicians per 100.000 Population					,489**	,780**
Standardized Number of All Nursing per 100.000 Population						,919**

b) Pearson correlation for variables representing poor and rural/remote population (n=34). Significant at **.P<0,01 and * P<0,05 (2-sided).

	Percentage of rural poor population related to total population	Percentage of total poor population related to total population	Percentage of rural in overall poor population
Percentage rural population	,426*	,534**	,898**
Percentage of rural poor population related to total population		,953**	,628**
Percentage of total poor population related to total population			,620**

Supplement Table S4: Discrimination analysis of population-based clusters, detailed parameters.

a) Results of Classification

			Predicted group			
			3T population	Non-3T population	3T low poor population	Total
Original cluster	Number	rural & poor population	13	1	0	14
		non-rural & non-poor population	4	11	1	16
		rural & low poor population	0	1	3	4
	%	rural & poor population	92,9	7,1	,0	100,0
		non-rural & non-ppor population	25,0	68,8	6,3	100,0
		rural & low poor population	,0	25,0	75,0	100,0

b) Explained variance by functions

Eigenwerte

Funktion	Eigenwert	% der Varianz	Kumulierte %	Kanonische Korrelation
1	1,646[a]	78,7	78,7	,789
2	,446[a]	21,3	100,0	,555

a first two canonic functions were used for modelling

c) Discriminative significance of functions

Wilks-Lambda

Test of function(s)	Wilks-Lambda	Chi-Quadrat	df	Significance
1 bis 2	,261	38,249	12	,000
2	,691	10,514	5	,062

Supplement Table S5: Cluster annotation of provinces

Cluster	Province
3T regions Non-Poor	Kepulauan Riau
	West Jawa
	DI Yogyakarta
	Banten
	Bali
3T regions Poor	Aceh
	Lampung
	Nusa Tenggara Timur
	West Kalimantan
	Central Sulawesi
	South Sulawesi
	Southeast Sulawesi
	Gorontalo
	West Sulawesi
	Maluku
	North Maluku
	West Papua
	Papua
Non-3T regions	North Sumatera
	West Sumatera
	Riau
	Jambi
	South Sumatera
	Bengkulu
	Kepulauan Bangka Belitung
	Central Java
	East Java
	Nusa Tenggara Barat
	Central Kalimantan
	South Kalimantan
	East Kalimantan
	North Kalimantan
	North Sulawesi
Not clustered	DKI Jakarta

Supplement Table S6: Variance analysis of service usage indicators for PBI & Non-PBI groups related to infrastructure clusters (n=33 provinces)

a) Unifactorial ANOVA

	Square sum	df	Mean of squares	F	Significance
Utilization Index PBI (FKTP non-capitation/capitation)	,055	2	,027	2,960	,067
Utilization Index Non-PBI (FKTP non-capitation/capitation)	,006	2	,003	6,267	,005
Referral index of primary to secondary care PBI (non-capitation FKRTL/FKTP)	33,604	2	16,802	4,569	,019
Referral index Non-PBI (non-capitation FKRTL/FKTP)	348,828	2	174,414	3,651	,038
Referral index All (non-capitation FKRTL/FKTP)	422,700	2	211,350	10,970	,000
FKRTL Utilization Rate PBI	,074	2	,037	7,345	,003
FKRTL Utilization Rate	,566	2	,283	9,858	,001
FKRTL Utilization Rate All	,682	2	,341	20,709	,000

b) Multifactorial comparison (Scheffé-procedure). Significant at *.P<0,05

	Population Cluster		Mean difference	Std.-Deviation	Significance	95%-Confidence intervall	
						Lower limit	Upper limit
Utilization Index PBI (FKTP non-capitation/capitation)	3T regions Poor	Non-3T regions	,07503	,03641	,137	-,0187	,1688
		3T regions Non-Poor	,10055	,05056	,156	-,0297	,2307
	Non-3T regions	3T regions Poor	-,07503	,03641	,137	-,1688	,0187
		3T regions Non-Poor	,02552	,04961	,877	-,1022	,1533
	3T regions Non-Poor	3T regions Poor	-,10055	,05056	,156	-,2307	,0297
		Non-3T regions	-,02552	,04961	,877	-,1533	,1022
Utilization Index Non-PBI (FKTP non-capitation/capitation)	3T regions Poor	Non-3T regions	,02297*	,00852	,039	,0010	,0449
		3T regions Non-Poor	,03736*	,01183	,014	,0069	,0678
	Non-3T regions	3T regions Poor	-,02297*	,00852	,039	-,0449	-,0010
		3T regions Non-Poor	,01439	,01161	,473	-,0155	,0443
	3T regions Non-Poor	3T regions Poor	-,03736*	,01183	,014	-,0678	-,0069
		Non-3T regions	-,01439	,01161	,473	-,0443	,0155
Referral index PBI (non-capitation FKRTL/FKTP)	3T regions Poor	Non-3T regions	-1,71932	,72667	,077	-3,5906	,1520
		3T regions Non-Poor	-2,67275*	1,00915	,043	-5,2715	-,0740
	Non-3T regions	3T regions Poor	1,71932	,72667	,077	-,1520	3,5906
		3T regions Non-Poor	-,95343	,99028	,634	-3,5036	1,5967
	3T regions Non-Poor	3T regions Poor	2,67275*	1,00915	,043	,0740	5,2715
		Non-3T regions	,95343	,99028	,634	-1,5967	3,5036
Referral index Non-PBI (non-capitation FKRTL/FKTP)	3T regions Poor	Non-3T regions	-4,95093	2,61895	,185	-11,6952	1,7934
		3T regions Non-Poor	-9,13192	3,63701	,057	-18,4979	,2341
	Non-3T regions	3T regions Poor	4,95093	2,61895	,185	-1,7934	11,6952
		3T regions Non-Poor	-4,18098	3,56902	,511	-13,3719	5,0100
	3T regions Non-Poor	3T regions Poor	9,13192	3,63701	,057	-,2341	18,4979

		Non-3T regions	4,18098	3,56902	,511	-5,0100	13,3719
Referral index All (non-capitation FKRTL/FKTP)	3T regions Poor	Non-3T regions	-5,86080*	1,66329	,006	-10,1441	-1,5775
		3T regions Non-Poor	-9,71617*	2,30985	,001	-15,6645	-3,7678
	Non-3T regions	3T regions Poor	5,86080*	1,66329	,006	1,5775	10,1441
		3T regions Non-Poor	-3,85537	2,26668	,251	-9,6925	1,9818
	3T regions Non-Poor	3T regions Poor	9,71617*	2,30985	,001	3,7678	15,6645
		Non-3T regions	3,85537	2,26668	,251	-1,9818	9,6925
FKRTL Utilization Rate PBI	3T regions Poor	Non-3T regions	-,05034	,02685	,190	-,1195	,0188
		3T regions Non-Poor	-,14196*	,03729	,003	-,2380	-,0459
	Non-3T regions	3T regions Poor	,05034	,02685	,190	-,0188	,1195
		3T regions Non-Poor	-,09162	,03659	,058	-,1858	,0026
	3T regions Non-Poor	3T regions Poor	,14196*	,03729	,003	,0459	,2380
		Non-3T regions	,09162	,03659	,058	-,0026	,1858
FKRTL Utilization Rate Non-PBI	3T regions Poor	Non-3T regions	-,14819	,06423	,086	-,3136	,0172
		3T regions Non-Poor	-,39156*	,08919	,001	-,6212	-,1619
	Non-3T regions	3T regions Poor	,14819	,06423	,086	-,0172	,3136
		3T regions Non-Poor	-,24336*	,08753	,032	-,4688	-,0180
	3T regions Non-Poor	3T regions Poor	,39156*	,08919	,001	,1619	,6212
		Non-3T regions	,24336*	,08753	,032	,0180	,4688
FKRTL Utilization Rate All	3T regions Poor	Non-3T regions	-,19171*	,04862	,002	-,3169	-,0665
		3T regions Non-Poor	-,41958*	,06752	,000	-,5935	-,2457
	Non-3T regions	3T regions Poor	,19171*	,04862	,002	,0665	,3169
		3T regions Non-Poor	-,22787*	,06626	,007	-,3985	-,0572
	3T regions Non-Poor	3T regions Poor	,41958*	,06752	,000	,2457	,5935
		Non-3T regions	,22787*	,06626	,007	,0572	,3985
